# Attitudes towards mental health problems in a sample of United Arab Emirates’ residents

**DOI:** 10.1186/s43045-022-00255-4

**Published:** 2022-11-18

**Authors:** Gabriel Andrade, Dalia Bedewy, Ahmed Banibella Abdelmagid Elamin, Khadiga Yasser Abdelraouf Abdelmonem, Hajar Jamal Teir, Nour Alqaderi

**Affiliations:** Ajman University, Ajman, United Arab Emirates

**Keywords:** United Arab Emirates, Attitudes towards mental health, Family values, Gender

## Abstract

**Background:**

Mental health issues are still stigmatized in the United Arab Emirates (UAE), possibly due to cultural reasons. This implies that some mental health conditions (most notably depression and anxiety) are not properly treated, due to resistance to seek help. It is therefore important to assess attitudes towards mental health in the UAE and their possible association with other variables.

**Results:**

In this study with 201 participants, attitudes towards mental health were assessed. Results came out showing no association with gender, nationality, age, or emirate of residency. A correlation was found with traditional family values, and in turn, this adherence varied across genders, with males having higher levels.

**Conclusions:**

Mental health issues in the United Arab Emirates are still stigmatized, although some improvement is evident. Given that stronger values predict more negative attitudes towards mental health problem, it is recommended authorities in the United Arab Emirates attempt some cultural progressive reforms in order to remove the stigma from mental health problems.

## Background

In recent years, the United Arab Emirates (UAE) has made significant advances in scientific development. However, in the realm of the scientific treatment of mental illnesses, progress has been rather slow.

There is research suggesting that, as opposed to other areas of medicine, mental health treatment in the UAE is embedded in folk ideas that, albeit effective in some circumstances, largely become an obstacle to the adequate treatment of mental health conditions [15]. For example, in a report by Chowdhury, an Emirati person asserts that “in our (Emirati) culture, many tend to turn to healers for mental cases because they think it may have something to do with being possessed by bad devils, having weak faith in God or being affected by black magic” [9]. Likewise, there is evidence that in the UAE, there is the widespread belief that mental illness is contagious, and this prevents prospective physicians from developing a career in mental health services, thus rendering mental health facilities understaffed [5].

This is a concern for public health in the UAE, as data suggests that mental health issues are not properly treated (especially anxiety and depression). An important factor in this situation is the reluctance to seek professional help, due to the stigmatization of mental illness [23]. This has been widely documented in previous scholarship coming from the Middle East. For example, [31] note that in the Arab world, “a large diversity in the stigmatizing beliefs, actions and attitudes towards treatment of mental illness within the Arab population” is prominent, and “the influence of cultural variations on stigma should be explored further.” This article intends to do that, focusing on the United Arab Emirates.

This is a relevant issue in the wider Arab world, as it has had a major cultural impact on the region, especially considering the increasing Western influence that has taken place in the recent decades. For example, in a systematic reviews of mental health practices in the Arab world, Al-Krenawi [4] explains that “mental health patients in Arab countries tend to express their psychological problems in terms of physical symptoms, thereby avoiding the stigma attached to mental illness.” This is a problem that is compounded by the complexities of modern life in nations that are still developing. Al Krenawi also notes that mental health patients in Arab countries “also tend to underutilize mental health services and to hold negative attitudes toward formal mental health services.” This affects particularly women, as Okasha explains that in the Arab world, “women, in particular, are more likely to suffer various disorders including depression, anxiety disorders, somatization, and eating disorders” [24].

It is important to explore more rigorously what variables are associated with stigmatization of mental illness in the UAE. Particularly, there is a need to explore the attitudes towards mental health problems, so as to better inform awareness efforts. In this study, we assessed attitudes towards mental health in the UAE population, and their association with 3 important demographic variables in this nation: age, gender, emirate of residence, and nationality.

Despite its rapid modernization program, the UAE still strongly adheres to traditional Arab and Islamic values, particularly in relationship to the family [18, 29]. In many countries, mental illness stigma is associated with traditional family and religious values [25]. In this study, we also assessed the association of family values and attitudes towards mental health in the UAE.

This is also a constant found throughout much of the Arab world, as previous literature has widely documented it. For example, in a study by Thomas et al. [26], it is asserted that “notably, traditional healers distinguished between biomedical illness and states they attributed to demonological or metaphysical causes. The Islamic spiritual narrative was central to discussions of etiology, intervention and outcome. Greater integration of traditional healers within the UAE's mental health-care services would, in many cases, improve patient experience and outcomes.” Given the cross-cultural experience with Western countries and the significant migration of Arab populations to North America, mental health practitioners in Western countries are beginning to understand the need to adapt to the specific cultural conditions of Arabs when providing mental health services. As Erickson and Al-Timimi [11] explain, “to effectively address the needs of Arab Americans, mental health professionals need to be aware of their own biases and misperceptions regarding Arab Americans, have an accurate understanding of Arab cultural and sociopolitical backgrounds, and be able to identify culturally appropriate interventions for use with Arab American clients. This article reviews common stereotyped beliefs many America have about Arab Americans and the negative impact these stereotypes can have on the development of a positive Arab American ethnic identity.”

To the extent that this study seeks to assess the attitudes towards mental health in the United Arab Emirates, it aims to make a contribution to improve the provision of mental health services in the region.

## Methods

The aim of the study was to assess attitudes towards mental health problems in the sample. Research protocols were approved by Ajman University’s Institutional Review Board, in order to comply with ethical requirements.

Sample size was calculated on the basis of Slovin’s formula, assuming a population of 10,206,508 (as per the most recent census [28]), a margin of error of 5%, and a confidence level of 95%. The calculation came down to 385 individuals. Inclusion criteria were living in the UAE, and exclusion criteria were not being proficient in English.

Non-probabilistic sampling was used (on the basis of willingness to answer a long survey) for recruitment. Three-hundred eight-five individuals were approached in various settings: university campuses, malls, public spaces, and via email communication. Depending on participants’ preferences, three options were used to complete the surveys. For those individuals approached in physical settings, surveys were completed orally (researchers recorded their answers), or participants scanned a QR code on their phones, and in turn, this led to the survey. For those individuals receiving email invitations, a link was sent. Answers to the survey were stored in Microsoft Forms, and answers remained anonymous. Response rate was 57.44%; 201 participants (127 females, 74 males; mean age: 24.6, s.d. 8.61) returned surveys with complete answers.

The first part of the survey asked demographic information (age, gender, nationality, emirate of residence). Nationality was clustered in three groups, as reflected in UAE society: Arabs, South Asians, and rest of the world. Emirate of residence was clustered in two groups: northern emirates (Ajman, Fujairah, Sharjah, Ras Al Khaimah, Umm Al Quwain) and southern emirates (Dubai, Abu Dhabi).

The second part of the survey was the attitudes towards mental health problems scale (ATTMHP). This is a questionnaire that assesses subjects’ attitudes towards mental health problems [16]. It is made up of 35 items. Items reflect attitudes towards community views (e.g., “My community sees mental health problems as something to keep secret”), internal shame factors in a hypothetical situation of mental health problems (e.g., “I would blame myself for my problems”), and reflected shame factors (e.g., “I would worry that I would be letting my family’s honour down”). Items are arranged on a Likert scale from 1 (completely disagree) to 5 (completely agree), with higher scores indicating higher negative attitudes towards mental health problems. The questionnaire was administered in English; Cronbach’s alpha was 0.963, indicating excellent internal reliability. The ATTMHP scale has been validated in various cultural settings [8].

The third part of the survey was the family values scale. This is a questionnaire that assesses subjects’ adherence to traditional family values [6]. It includes 14 items such as “Mother’s place is at home” and “Problems should be resolved within the family.” Items are arranged on a Likert scale from 1 (completely disagree) to 5 (completely agree), with higher scores indicating higher adherence to traditional family values. The questionnaire was administered in English; Cronbach’s alpha was 0.896, indicating good internal reliability. The family values scale has been validated in various cultural settings [7].

ATTMHP and family values scale were analyzed for their association with demographic variables (nationality, gender, emirate of residence, and age). To do so, ANOVA (for nationality), *t*-tests (for gender and emirate of residence), and Spearman’s coefficients (for age) were done. Assumptions for parametric tests were verified, and when the assumptions were not met, nonparametric alternatives were used (Mann-Whitney *U*-test and Kruskal-Wallis test).

Spearman coefficients for correlation of family values scale with ATTMH were calculated.

A multivariable linear regression model was constructed with Jamovi software. Dependent variable was ATTMHP, and independent variables were gender, age, family values scale, emirate of residence, and nationality group (divided in two dummy variables, “South Asians,” “rest of the world”).

Statistical significance was placed at *p* < 0.05.

## Results

Descriptive results split by demographic variables (nationality, gender, emirate of residence) are presented in Table 1. For nationality, Arabs were the most represented group in the sample (71.1%), followed by South Asians (15.9%) and citizens from nations and from the rest of the world (12.9%). A total of 59.2% were residents from the northern Emirates, 40.8% were residents from the southern Emirates. A total of 36.8% were male, and 63.2% was female; mean age was 24.6.

**Table 1:** Descriptive statistics

	Representation in the sample	Age		ATTMHP			Family values		
Nationality		Mean	s.d.	Mean	Median	s.d.	Mean	Median	s.d.
Arabs	143 (71.1%)	24.4	8.94	98.6	98	30.6	51.2	53	11.4
South Asians	32 (15.9%)	24.9	7.73	109	109	25.7	51.6	49.5	10.5
Rest of the world	26 (12.9%)	25.4	8.07	102	104	25.7	53.2	53	7.39
**Gender**									
Male	74 (36.8%)	26.4	10.2	104	102	28.5	53.3	56	12.9
Female	127 (63.2%)	23.6	7.37	98.4	99	31	50.5	52	9.29
**Emirate of residence**									
Northern Emirates	119 (59.2%)	25.3	9.34	103	102	29.9	49.6	51	12.4
Southern Emirates	82 (40.8%)	23.7	7.39	96.9	99.5	30.3	52.9	54	9.43

Associations with demographic variables for ATTMHP are presented in Table 2. No demographic variable is statistically significant in its association with ATTMHP. The variable that came closest to statistical significance was emirate of residence, but its *p*-value (0.155) was still far from the cutoff alpha value.

**Table 2:** Demographic associations of ATTMHP

Variable	Statistic	***p***-value
Gender (*t*-test)	−1.38	0.169
Emirate of residence (*t*-test)	−1.47	0.143
Nationality (one-way ANOVA)	1.94	0.155
Age (Spearman’s coefficient)	0.046	0.516

Associations with demographic variables for family values are presented in Table 3. Gender has a statistically significant association with family values scale with males having higher scores (median = 66) than females (median = 52). Emirate of residence came close to statistical significance, but it was still removed from the cutoff alpha value.

**Table 3:** Demographic associations of family values scale

Variable	Statistic	***p***-value
Gender (Mann-Whitney *U*)	3716	0.0013*
Emirate of residence (Mann-Whitney *U*)	4169	0.08
Nationality (Kruskal-Wallis)	0.255	0.88
Age (Spearman’s coefficient)	−0.015	0.83

Spearman’s coefficient for the correlation between ATTMHP and family values is 0.182, *p*-value 0.01. This indicates a weak but statistically significant correlation. The multivariable linear regression model is presented in Table 4. In this model, *R* is 0.287, *R*^2^ is 0.0825, and *p*-value is 0.01. This model therefore indicates that 8% of the variation within the “attitudes towards mental health” can be attributed to the conjunction of family values, gender, age, nationality, and emirate of residence. Out of all predictors, only the variable “family values” is statistically significant. When the nonsignificant predictors are removed from the model and only “family values” are preserved, the resulting regression equation is *y* = 65.59 + 0.68 (x).

**Table 4:** Model coefficients — attitudes towards mental health problems

Predictor	Estimate	SE	***t***	***p***	Stand. estimate
Intercept	65.937	11.394	5.787	< .001*	
Family values	0.634	0.197	3.224	0.001*	0.2272
Gender	3.596	4.533	0.793	0.429	0.0577
Age	−0.152	0.246	−0.618	0.537	−0.0434
South Asians	8.89	5.905	1.506	0.134	0.1081
Rest of the world	2.077	6.275	0.331	0.741	0.0232
Emirate of residence	4.673	4.309	1.084	0.279	0.0764

*p < 0.05

## Discussion

As per the multivariable regression model of the independent variables under consideration, only family values have a statistically significant effect on attitudes towards mental health. This is somewhat encouraging, as it defies the expectation of gender differences in the UAE. Although there remains much to be done in favor of women empowerment in this nation, the UAE has made significant advances in that regard. Over the last decade, women in the UAE have moved towards better positions in society, and this has narrowed many gender gaps. In turn, this may reflect the lack of any meaningful statistical difference when it comes to attitudes towards mental health. While the effects of the Covid-19 pandemic have had an impact on women’s mental health in the UAE, it can still be taken as an encouraging sign that women are not more likely than men to have stigmatizing and negative attitudes towards mental health.

Likewise, it is also an encouraging finding that there are no statistically significant differences in attitudes towards mental health across nationality groups. Traditionally, for both Arabs [12] and South Asians [14], research has shown that there are cultural factors conditioning members of those communities to have demeaning attitudes towards mental health problems. Yet, in the UAE, this pattern may be changing (as suggested by the results of this study), to the extent that as this nation has accelerated a program of modernization, more liberal attitudes have been embraced across many areas of social life, and consequently, neither Arabs nor South Asians are likely to have more demeaning attitudes towards mental health than residents from other regions of the world, especially those coming from Western nations.

While the southern emirates (Dubai, Abu Dhabi) have been modernized at a much faster pace than their northern counterpart (Ajman, Fujairah, Sharjah, Umm Al Quwain, Ras Al Khaimah), it is also noteworthy than in this study, there are no statistically significant differences across emirates when it comes to attitudes towards mental health. There is much mobility across emirates, and commuting is quite common [17]. In this regard, while emirates may vary in terms of urban development, place of residence has no effect on attitudes towards mental health.

There is a correlation between attitudes towards mental health and family values. The correlation is weak, but it is statistically significant, so as to still warrant some attention. Despite its rapid modernization, UAE social life still preserves tribal structures and prominence of kinship-based relations [13]. Traditional family values are given priority by both nationals and migrants [21]. To the extent that as per traditional family values honor plays a major role (especially in Arab societies) [10], mental health issues are more likely to be perceived as threatening the integrity of the family, given that mental health stigma affects the reputation of the family involved. Consequently, as the results of this study suggest, a stronger adherence to family values in the UAE serves as a predictor of demeaning attitudes towards mental health, albeit with a high degree of variability.

Given that adherence to conservative family values serves as a predictor of attitudes towards mental health issues, it is also important to examine how family values vary across nationality, emirate of residence, and gender. In this study, emirate of residence and nationality were not found to be predictive of adherence to family values.

This is somewhat surprising, given that it has been traditionally assumed that both Arabs and immigrants from South Asian countries have more robust conservative family values than persons coming from more westernized countries [3, 30]. This surprising result can be taken as evidence of the optimal assimilation and integration efforts of the UAE government, to the extent that residents of all nationalities are properly integrated to society, and consequently, they exhibit uniform patterns of family structure and values, thus building a form of “melting pot” integrated society in the UAE [20].

Likewise, the fact that no significant differences in family values are found across emirates may be a sign of the successful standardization of all seven emirates in terms of government programs [1]. As per modernization theory in sociology [22], when areas of varying degrees of modernization are influenced by rapid development, their family structure undergo rapid, uniform change, thus forming areas with similar patterns of family values. The results of this study provide confirmation of that hypothesis.

Interestingly, in terms of family values, a statistically significant difference was found across genders, with males presenting higher adherence to traditional family values. This finding coheres with results from previous research on gender relations in the UAE. Despite the significant advances in women empowerment and the great efforts put forth by UAE authorities to offer women greater opportunities in labor and education [2], patriarchal cultural attitudes persist [27], and it is notoriously difficult for governments to legislate such matters. Consequently, given the prevailing patriarchal attitudes, it is expected that traditional family values are more strongly defended by men (as confirmed by this study), although there is reason to believe that this may begin to change in the near future, as the rapid modernization pace in the UAE would ultimately promote cultural changes [19].

## Conclusions

The nature of this study has been preliminary, and some limitations ought to be considered. While the sample was sufficiently large so as to warrant statistically significant results, future studies must include larger samples in order to reach more robust conclusions. Likewise, this study was somewhat unbalanced in terms of gender distribution, as women made up 63% of respondents, when in fact the UAE’s population is majority male (due to the influx of labor migrants in the construction realm).

Nevertheless, some important conclusions can be obtained from this preliminary study. First, the results show that in the UAE, attitudes towards mental health problems are still considerably negative, and more works need to be done to remove the stigma from seeking psychological counseling or psychiatric help. Second, attitudes towards mental health have some association with adherence to traditional family values, and these traditional values are more likely to be upheld by males than by females.

An important finding of this study is that nationality and emirate of residence seem to play no role in any variance of attitudes towards mental health in the UAE. Consequently, public health authorities do not need to focus on specific national groups or emirates but, rather, must come up with strategies that encompass all of society in order to remove stigma from mental health problems.

Nevertheless, gender is an important variable to consider. For while there is no statistically significant gender difference when it comes to attitudes towards mental health, there is such a difference when it comes to adherence to traditional family values. Given that a stronger adherence to traditional family values predicts more demeaning attitude towards mental health (and in this study men had stronger adherence to traditional family values), public health officials ought to address gender roles associated with traditional culture, so as to promote less rigid family values (while preserving the positive aspects related to family), in the hope that such an initiative may help in removing the stigma that is still associated with mental health issues.

Further studies must consider additional variables and their possible association with attitudes towards mental health. Most notably, income and educational levels must be taken into account, given that they may be predictors of attitudes towards mental health.

## Data Availability

Data is available upon reasonable request.

## Abbreviations

UAE: United Arab Emirates
ATMHP: Attitudes towards mental health problems
ANOVA: Analysis of variance
